# Characterizing and predicting ccRCC-causing missense mutations in Von Hippel-Lindau disease

**DOI:** 10.1093/hmg/ddad181

**Published:** 2023-10-26

**Authors:** Adam Serghini, Stephanie Portelli, Guillaume Troadec, Catherine Song, Qisheng Pan, Douglas E V Pires, David B Ascher

**Affiliations:** School of Chemistry and Molecular Biosciences, Chemistry Building 68, Cooper Road, The University of Queensland, St Lucia, QLD 4072, Queensland, Australia; School of Chemistry and Molecular Biosciences, Chemistry Building 68, Cooper Road, The University of Queensland, St Lucia, QLD 4072, Queensland, Australia; School of Computing and Information Systems, University of Melbourne, Melbourne, VIC 3010, Australia; School of Computing and Information Systems, University of Melbourne, Melbourne, VIC 3010, Australia; School of Chemistry and Molecular Biosciences, Chemistry Building 68, Cooper Road, The University of Queensland, St Lucia, QLD 4072, Queensland, Australia; Computational Biology and Clinical Informatics, Baker Heart and Diabetes Institute, 75 Commercial Road, Melbourne, VIC 3004, Australia; School of Computing and Information Systems, University of Melbourne, Melbourne, VIC 3010, Australia; Computational Biology and Clinical Informatics, Baker Heart and Diabetes Institute, 75 Commercial Road, Melbourne, VIC 3004, Australia; School of Chemistry and Molecular Biosciences, Chemistry Building 68, Cooper Road, The University of Queensland, St Lucia, QLD 4072, Queensland, Australia; Computational Biology and Clinical Informatics, Baker Heart and Diabetes Institute, 75 Commercial Road, Melbourne, VIC 3004, Australia

**Keywords:** Von Hippel-Lindau, VHL, clear cell renal cell carcinoma, ccRCC, machine learning

## Abstract

Background: Mutations within the Von Hippel-Lindau (VHL) tumor suppressor gene are known to cause VHL disease, which is characterized by the formation of cysts and tumors in multiple organs of the body, particularly clear cell renal cell carcinoma (ccRCC). A major challenge in clinical practice is determining tumor risk from a given mutation in the VHL gene. Previous efforts have been hindered by limited available clinical data and technological constraints. Methods: To overcome this, we initially manually curated the largest set of clinically validated VHL mutations to date, enabling a robust assessment of existing predictive tools on an independent test set. Additionally, we comprehensively characterized the effects of mutations within VHL using *in silico* biophysical tools describing changes in protein stability, dynamics and affinity to binding partners to provide insights into the structure-phenotype relationship. These descriptive properties were used as molecular features for the construction of a machine learning model, designed to predict the risk of ccRCC development as a result of a VHL missense mutation. Results: Analysis of our model showed an accuracy of 0.81 in the identification of ccRCC-causing missense mutations, and a Matthew’s Correlation Coefficient of 0.44 on a non-redundant blind test, a significant improvement in comparison to the previous available approaches. Conclusion: This work highlights the power of using protein 3D structure to fully explore the range of molecular and functional consequences of genomic variants. We believe this optimized model will better enable its clinical implementation and assist guiding patient risk stratification and management.

## Introduction

Von Hippel-Lindau (VHL) disease has the inheritance pattern of an autosomal dominant disease, with an estimated prevalence of up to 1 in 36 000 individuals among the general population [1]. This condition can lead to the development of several pathological conditions including cysts and tumors within multiple organs of the body, such as the liver, kidneys, retina, brain and spinal cord [2]. Common oncological manifestations of VHL disease include Clear Cell Renal Cell Carcinoma (ccRCC), pheochromocytoma (PPC), and hemangioblastomas within the central nervous system and retina, though one of the most frequently observed tumor pathologies of VHL disease is ccRCC [3, 4]. VHL disease is brought about through the acquisition of inactivating mutations in both alleles of the VHL gene within a single cell, with approximately 52% of such mutations classified as missense mutations [5]. VHL is classified as a tumor suppressor gene, therefore mutations that inactivate this gene predispose VHL patients to neoplasm generation and subsequent tumor formation [6].

Multiple isoforms of the VHL protein are generated through alternative splicing mechanisms, with the isoform of concern regarding VHL disease being the canonical pVHL isoform 1 protein product (pVHL30). This is due to its role in regulating HIF1α (Hypoxia-inducible factor 1-alpha), as well as other proteins involved in tumor suppression [7]. The pVHL30 consists of unstructured regions and two structured domains: the alpha and beta domains. The alpha domain is responsible for forming a complex with elongin B and elongin C, while the beta domain interacts with hydroxylated prolines of HIF-1α (Supplementary Fig. 1). The physiological significance of the pVHL30 structure varies along different regions of the protein, with the N-terminal domain being shown to be very disordered where augmentations are less likely to impact the molecular functions of the protein, while the alpha and beta domains exhibit a large degree of orderliness and hence are much more likely to play a critical role in the tumor suppressor functions of the protein [8–10].

The VHL protein reduces tumor formation via modification of multiple chemical signaling pathways, the most well studied of which being its role in HIF-1α regulation. A complex is formed between pVHL, Elongin B and Elongin C, in the cytoplasm of the cell. Via the recruitment of the E3 ubiquitin ligase complex, VHL facilitates the ubiquitination and subsequent proteasomal degradation of HIF-1α under normal physiological conditions [11, 12]. However, in hypoxic conditions or in the event that pVHL becomes compromised (as observed in VHL disease), the binding of pVHL to HIF-1α does not occur. This leads to the accumulation of HIF-1α within the cell’s cytoplasm and its translocation to the nucleus, where it acts as a transcription factor targeting and upregulating hypoxia-responsive genes. This includes genes involved in anaerobic energy metabolism (e.g. *GLUT1*), regulating oxygen transportation (e.g. *EPO*), and initiating angiogenesis (e.g. *VEGF*), resulting in a higher probability of the formation of neoplasms and subsequent tumor formation [13–16]. pVHL also acts to support the activation of the p53 tumor suppressor protein [17, 18]. However, the interactions between VHL and p53 are not well defined.

Innovations in the field of computational biology have provided highly accurate methodologies to assess the impact of mutations on the risk of disease development [19, 20]. We have previously interrogated this concept using structure-based statistical and machine learning approaches [21, 22] in genetic diseases [23–30] and cancers [31–36], as well as cancer and anti-microbial drug resistance [37–45]. Within the VHL space, we previously developed a machine-learning model, Symphony, capable of predicting the risk of ccRCC development upon missense mutation [46]. While Symphony exhibited good accuracy and specificity when it was first published, it primarily considered effects of mutations on protein thermal stability. The increased amount of publicly available data regarding VHL mutation pathogenicity and advancement of molecular feature generation tools since its inception presents the opportunity to evaluate these previous models, and integrate a more comprehensive picture of molecular and functional consequences of mutations to improve predictive performance. In this work, we built upon findings from Symphony using novel techniques and current clinical data to develop an up-to-date ccRCC development predictor for missense mutations in the VHL gene. Our new tool offers invaluable applications clinically, particularly in prioritizing patient monitoring protocols according to cancer risk.

## Results

### Data curation

We obtained ccRCC and non-ccRCC VHL mutations from the Symphony database, which consisted of a total of 121 missense mutations, and were used for the training of our model. Additionally, our literature search from 96 unique articles identified a further 92 mutations, which were not considered during the development of Symphony, and were used as a non-redundant test set. Overall, our data consisted of 142 ccRCC-causing mutations and 71 non-ccRCC causing mutations, (Supplementary Table 1).

**Figure 1:**
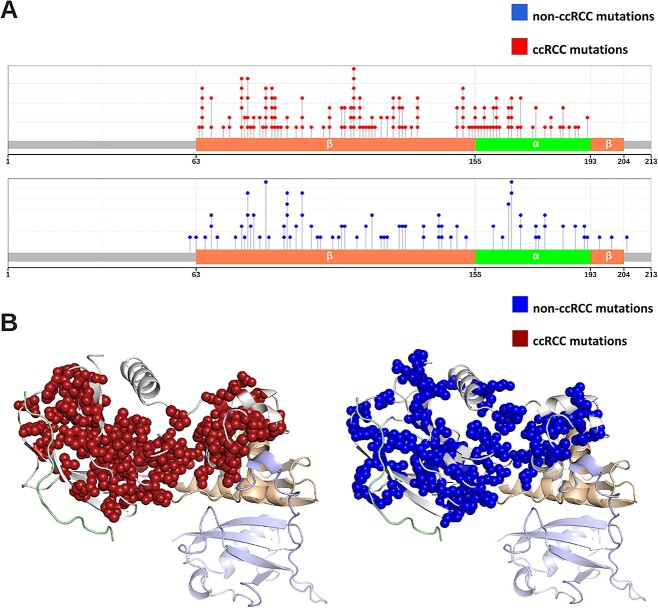
pVHL30 mutation distribution by phenotype. Illustration of the ccRCC and non-ccRCC mutations in the primary sequence of pVHL30 (A), and its 3-dimensional structure (B). Mutations of both phenotypes are concentrated in the structured regions of the alpha and beta domains, and are mostly absent from the unstructured regions of pVHL30.

In observing the distributions of both classes of mutations in a linear (lollipop plot, Fig. 1A) and 3-dimensional representation (Fig. 1B), we observed that mutations are mainly situated within the structured regions of the VHL protein, with multiple overlaps in phenotypes within the same loci. Additionally, there does not seem to be any clear discernible pattern of differentiation between the two phenotypes and mutation location from these illustrations alone.

**Figure 2:**
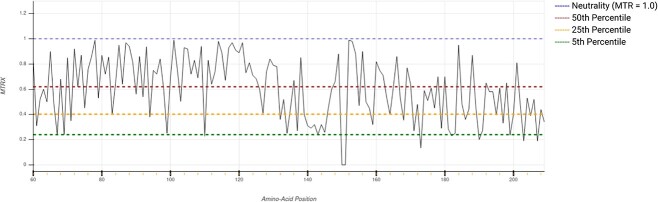
MTR scores of pVHL30 amino-acid position calculated by MTR3D. Horizontal lines show gene-specific MTR percentiles 5th, 25th, 50th, and neutrality.

In order to further investigate the relationship between the mutation position and ccRCC development, we used MTR3D to estimate missense intolerance of pVHL30. MTR3D is a computational tool that estimates the deleteriousness of a missense variant, by utilizing datasets of observed variation within the population to calculate the Missense Tolerance Ratio (MTR). The MTR value is used to identify regions of a protein that are under a selection pressure and that are intolerant to genetic changes [46–48]. Average MTR scores of alpha and beta domains are 0.52 and 0.65, respectively, suggesting the alpha domain is more tolerant to mutations than the beta domain (Fig. 2). Interestingly, however, there are two small regions of the beta domain where no ccRCC-causing mutations have been recorded (residues 137–148 and 193–203), which map onto a linker region and alpha helix of the domain. This absence of ccRCC-causing mutations is reflected in a lower average MTR score for these regions of 0.44 compared to that of the rest of the beta domain, which has an average score of 0.72.

### Evaluation of current pathology prediction tools

In order to allow for a direct comparison of our final model with other known tools, a literature-curated, non-redundant dataset was used as a benchmarking dataset. Prediction tools tested included the VHL-specific tool Symphony, and more general disease predictors, Polyphen-2 and SIFT (see Table 1). PolyPhen-2 (Polymorphism Phenotyping v2) predicts the functional and stability changes of a protein from single nucleotide polymorphisms [49] and SIFT (Sorting Intolerant From Tolerant) is a sequence homology-based tool that predicts a change in the phenotypic effect from a single amino acid change [50]. This analysis demonstrated the poor performance of the aforementioned tools in distinguishing between ccRCC and non-ccRCC causing mutations on this newly curated data set, demonstrating the need for a novel and optimized model to assist on VHL patient risk stratification.

**Table 1:** Comparison of performance of prediction tools for ccRCC development from VHL missense mutations.

Performance metric	Symphony	SIFT	Polyphen-2
MCC	0.075	0.032	0.036

Each predictive tool was evaluated using the MCC metric, using the same non-redundant dataset.

### Statistical analysis

In order to further our understanding of VHL-mediated ccRCC, statistically significant (defined as *P*-value < 0.05) discrepancies of molecular and biophysical characteristics between ccRCC and non-ccRCC VHL missense mutations were identified through a Welch sample t-test.

**Figure 3:**
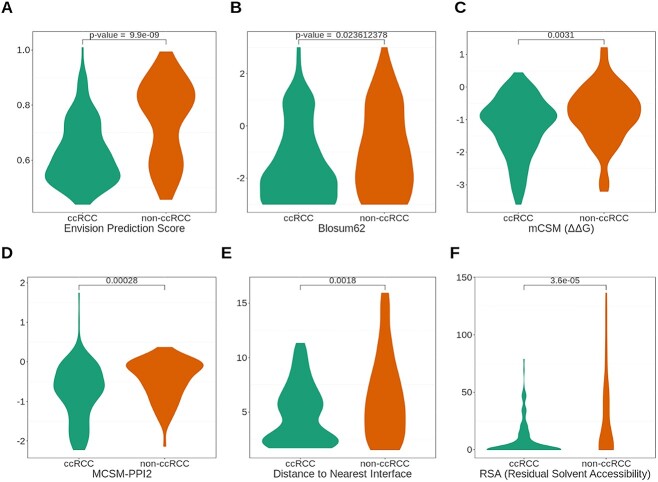
Comparison of key molecular features between ccRCC and non-ccRCC-causing mutations. Statistical significance was calculated using the Welch for the Envision Prediction score (A), Blosum62 (B), mCSM ΔΔG (C), mCSM-PPI2 (D), distance to nearest interface (E), and RSA (F).

Interestingly, of all the molecular features tested, the feature that displayed the highest statistical difference between the two phenotypes was generated from a stochastic gradient boosting model called Envision (*P*-value < 9.9E−9) (Fig. 3A). Envision predicts the extent to which a mutation is damaging for a protein compared to the wild-type, providing a quantitative predictor of molecular effect [51].

Furthermore, the likelihood of amino acid change is highly dependent on the disparities of physicochemical properties of the residues, such as residue pH and polarity, thus it was suspected that these features may differ between ccRCC and non-ccRCC mutations. Numerous features describing the evolutionary conservation and amino acid substitution probabilities were generated, and calculated using PAM and BLOSUM matrices, which describe the rate at which amino acids are substituted for other amino acids. Analysis of the sequence-based features revealed noticeable disparities between the two phenotypes, with higher scores observed in the non-tumor causing mutations compared to that of the tumor causing mutations. For example Blosum62 (*P*-value = 2.36E−3) as seen in Fig. 3B, indicates that pVHL30 non-ccRCC mutations occur more frequently than ccRCC-causing mutations.

Additionally, the thermodynamic properties of a molecule have a significant influence on its behavioral properties. Protein stability, particularly in the structured regions of a protein, is typically necessary for it to carry out its normal biological function, and the greater destabilizing effect of a mutation the greater disturbances to its normal physiological function are expected. Several tools were used to predict the change in enthalpy of the mutated protein compared to that of the wild type (e.g. Dynamut2, mCSM-Stability). Using mCSM-Stability, it was observed that there was a significant difference in predicted Gibbs free energy change between ccRCC causing and non-ccRCC causing mutations (Fig. 3C), *P*-value = 3.10E−3, as shown previously, with ccRCC-causing mutations leading to larger destabilizing effects [52].

Often, cancerous mutations exert their pathogenic effects by disturbing critical protein-protein interactions necessary for normal physiological function. It is for this reason, we investigated the change in protein affinity for ccRCC and non-ccRCC mutations for all proteins within the VHL complex. This was accomplished using mCSM-PPI2, a computational tool used to predict the change in protein-protein affinity from missense mutations. Using the results from mCSM-PPI2, a larger decrease in protein affinity could be seen in ccRCC-causing mutations than non-ccRCC-causing mutations (Fig. 3D), *P*-value = 2.80E−4.

Moreover, the spatial arrangement and packaging of the amino acid residues within a protein has enormous ramifications on its structure and function. Thus, the relative location of the amino acid change within the VHL protein complex was investigated. Analysis of the distance of a missense mutation to the various interfaces in the VHL protein complex showed a tendency of cancer-causing mutations to be located in closer proximity to any of the protein interfaces within the VHL complex (Fig. 3E), *P*-value = 1.80E−3.

Another influential attribute of protein function is the solvent exposure of amino acid residue in a protein. Solvent exposure is an important factor in protein-protein interactions, recognition of protein fold and identification [53, 54]. Thus, examining the solvent exposure of different disease phenotypes may provide insight into the patterns of ccRCC mutations. Several different measures of solvent exposure are widely used, but the one of particular interest is the relative solvent accessibility (RSA), which is the distance between residue and closest surface water molecule. Examination of the RSA for residues within the VHL complex revealed ccRCC-causing mutations are localized closer within the protein core, while non-ccRCC mutations localized more toward the surface (Fig. 3F), *P*-value −3.60E−5.

### A new machine learning model to predict ccRCC-causing mutations

During model development, where we tested 11 different algorithms, Random Forest consistently outperformed alternative approaches. This was hence chosen as our final model, which showed a Matthew’s Correlation Coefficient (MCC) value of 0.44 under 10-fold cross-validation and on the blind test (Table 2), both of which were composed of data from clinical studies. This demonstrates the superior performance of our model compared to that of previous methods. Considering other metrics (Table 2) we also observed an AUC (Area Under Precision Recall Curve) of 0.71 and 0.77 for the cross validation and blind test, respectively, and accuracy scores of 0.73 (cross validation) and 0.81 (blind test). This demonstrates the robustness of our model. Using this model, predictions were calculated for each possible missense mutation within the VHL experimental crystallographic structure (Supplementary Table 2). A total of 1306 ccRCC-causing mutations and 1544 non-ccRCC-causing mutations were predicted, with no clear pattern of distribution between the two phenotypes along the 2D sequence of the VHL protein.

**Table 2:** Performance matrices of random forest model for 10-fold CV and blind test set.

Validation method	F1-score	Accuracy	AUC	MCC
10-fold CV	0.72	0.73	0.71	0.44
blind-test	0.80	0.81	0.77	0.44

## Discussion

VHL is a genetic disease that predisposes an individual to cysts and tumor formations within the retina, central nervous system, adrenal glands and kidneys. It is caused by the loss of both functional VHL alleles within a single cell, most often a result from missense mutations. One of the most common tumor types caused by VHL disease is ccRCC. Knowledge of the likelihood of a VHL mutation resulting in ccRCC development can aid clinicians in developing more effective screening strategies. Previous work attempted to resolve this issue by developing a machine learning model, named Symphony, to predict the development of ccRCC from missense mutations. However the performance of this model was constrained by the limited amount of clinical data and computational tools available at its time of development. It is for this reason, this study aimed to produce an up-to-date ccRCC development predictor for missense mutations in the VHL gene. This was achieved by curating the largest curated VHL missense mutation data set to date.

Data regarding ccRCC and non-ccRCC causing mutations was collected from the Symphony database, as well as a literature review of clinical studies. While these were used as the primary source of data in this study, other cancer-dedicated databases, like TCGA and COSMIC are also publicly available, and present clinically relevant cancer data to varying degrees. Because of the data heterogeneity these databases may have added to our curated set, we have chosen to forgo these sources for this current work. This ensured that our work, which focused on ccRCC mutations in VHL, was as clinically relevant as possible. Considering the multiple cancers VHL is involved in, however, we do not exclude the utility of these other databases in future expansions of this work. Predictably, analysis of said curated data revealed mutations concentrated in structured regions of pVHL30. However, no clear pattern distinguishing our classes could be observed when assessing mutation distribution, where mutations from both phenotypes were observed to overlap across pVHL30 domains. Meanwhile, the MTR results indicated that the alpha domain of the protein is more tolerant to mutations than the beta domain.

**Figure 4:**
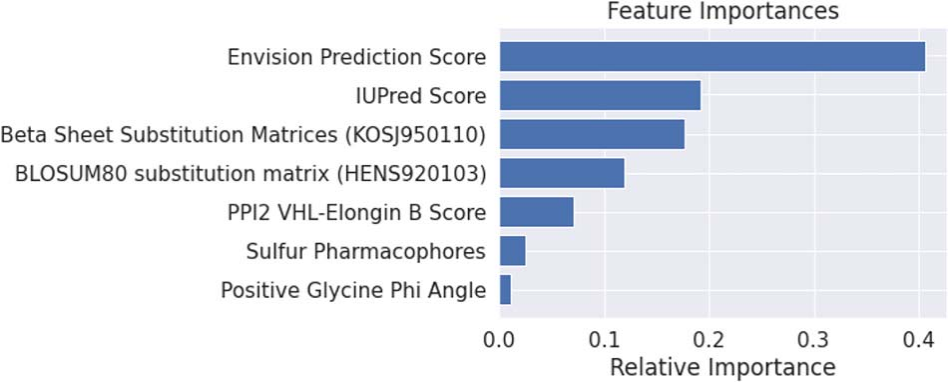
Feature importance of Random Forest model. Examination of the relative importance of each feature in the Random Forest model, reveals that the Envision prediction score to be the most significant feature in the models prediction calculations by a substantial margin.

The biophysical attributes of VHL mutations were then examined, in order to provide insight into the molecular characteristics that contribute to tumor formation. For this purpose, statistical analysis of VHL mutations was performed using molecular features generated from the experimental crystallographic structure of VHL. This revealed multiple intriguing differences between ccRCC and non-ccRCC mutations. One such difference is the Envision prediction score, which quantifies the deleteriousness of a mutation. This score was shown to be a powerful predictor of ccRCC development. In addition to showing the highest statistically significant average difference between ccRCC and non-ccRCC mutations among all the different molecular features investigated, the Envision prediction score also possessed the highest relative importance value (Fig. 4) in the Random Forest machine learning model generated. This demonstrates the utility of the Envision score in predicting the impact of missense mutations.

In our analysis, we observed that ccRCC-causing mutations not only correlated to protein destabilization, but also showed lower amino acid conservation scores in comparison to non-ccRCC mutations. Additionally, the location of the mutated amino acid residues contributed to the likelihood of ccRCC development, as ccRCC-causing mutations were more likely to be located in buried protein regions, as well as being positioned closer to the interface with other proteins in the VHL complex.

Previous attempts have been made to predict ccRCC mutations, which integrated machine learning tools to predict ccRCC causing mutations. However, the Symphony model is limited by a small dataset and a reduced number of tools for molecular feature generation. This study overcomes these previous limitations by constructing a machine learning model, utilizing additional clinical data and current computational tools, as well as using an independent clinical testing dataset for evaluation purposes.

The model developed in this study integrates data regarding the biophysical, spatial and evolutionary differences between the wild type and mutated residues. When compared using the same dataset, our model outperformed all alternative methods, including Polyphen-2 and SIFT, which are widely used clinically to prioritize patients according to their mutation profiles.

Despite its high performance, our model also contains limitations. Due to the limitations of the crystallographic structure used for the VHL complex, the model can only generate missense mutation predictions for 150 of 213 amino acid residues in pVHL30. However, the residues that are beyond the scope of the model’s prediction are located within unstructured regions of the protein and thus are unlikely to produce pathological consequences upon mutation. In fact, there has been very few published instances of ccRCC-causing mutations occurring within these unstructured regions.

A limitation to the performance of the model is the absence of p53 interaction data used to train the model. It is well established that VHL is involved in the regulation p53, a tumor suppressor protein with a significant role in cancer prevention and development in the body. Due to lack of structural data regarding the interaction with VHL and p53, this information was not integrated into the training of the model. It is likely that with this data, the model would have greater accuracy and prediction capabilities, particularly because of the strong connection between both these genes and clinical cancer manifestation.

The loss of both functional VHL alleles within a single cell predisposes an individual to tumor formation occurring from said cell, with one of the most common types of tumor developing being ccRCC. Investigation into the biophysical, structural and evolutionary conservation characteristics of VHL mutations, showed statistically significant disparities between mutations causing ccRCC and those that do not. Furthermore, a machine learning model was constructed to accurately predict the development of ccRCC from a missense mutation within the VHL protein, which considerably outperformed previous VHL-specific and general disease classifiers.

## Materials and Methods

### Data collection

The primary objective of this study was to predict the physiological consequences of a missense mutation within the VHL gene. For this purpose, VHL missense mutation data was collected from the Symphony database, consisting of ccRCC-causing and non-ccRCC-causing mutations, of which, the latter may include the clinical manifestation of other cancers like hemangioblastoma and paraganglioma. Additionally, mutational ccRCC VHL data was gathered from the ClinVar database [55] (accessed August 2022) (Supplementary Table 1). The clinical involvement and labeled phenotype of mutations listed in ClinVar were verified by the literature. Only data produced from VHL patients were retained for analysis and model generation. Moreover, mutations not present in the ClinVar database but observed in patients within the clinical literature were also retained. In addition, a general literature review of clinical studies involving VHL patients was also carried out using key-word searches in the PubMed database. This data was then divided and used for the training and subsequent testing of the machine learning models (Supplementary Table 3).

### Feature generation

There are numerous computational methodologies that aim at describing different aspects of effects of mutations on protein structure, function and interactions, ranging from molecular interactions, general physicochemical properties and 3-dimensional shape, to protein-level stability changes and effects on interactions with other molecules [56–62]. We used the aforementioned descriptors to represent missense mutations within the VHL gene and protein, for the purposes of generating molecular features. This is based on the premise that the degree to which mutation interatomic interactions deviate from wild type can be indicative of the pathogenicity mechanism of the mutated protein.

Molecular features were calculated using an experimental crystallographic structure describing VHL in a protein complex with Elongin B, Elongin C and HIF-α (PDB ID: 1LM8). Preprocessing was performed on the structure preceding feature generation using the software tool named Maestro, and involved the removal of water molecules and metal atoms, correction of missing residue atoms and addition of missing residues present in pVHL30.

This study implements the use of multiple *in silico* methods in order to generate numerical representations of the pVHL30 from each possible VHL missense mutation. A total of 314 features were generated (Supplementary Table 4), which can be broadly grouped into 6 different categories:

1. Graph-based signatures: In which the protein residue environment was represented by a graph, to model atoms and physicochemical interactions that are in close proximity [58].
2. Effects on thermodynamics: DUET [57], mCSM-Stability [58] and Dynamut2 [59, 61] were used to predict changes in protein stability as a change in Gibbs free energy change (ΔΔG, in Kcal/mol).
3. Structural properties: several measures of the amino acids position relative to the VHL protein complex were calculated. These included Residue Depth (the average distance of the atoms of a residue from the solvent accessible surface) and the Residue solvent accessibility, or RSA (a measure of how exposed or buried a residue is in the 3D structure of a protein) generated using BioPython, distance to the protein interface, and backbone phi and psi angles.
4. Affinity changes: predictions generated using machine learning tools, mCSM-PPI and mCSM-PPI2 [60], regarding the effect of mutations on binding affinity between VHL and its respective interacting proteins HIF-1α, Elongin B and Elongin C. In each case, values for mutations lying beyond 10 Å of the protein-protein interface were masked to solely retain direct effects on affinity.
5. Interatomic Interactions: Arpeggio was used to calculate several different subtypes of wild type interatomic interactions in a protein, as well as their changes upon mutation [56].
6. Sequenced-based features: The AAindex database, which contains physico-chemical and biochemical properties of amino acids, was used to produce molecular features of each substitution mutation [63]. Additionally, the BLOSUMs and PAMs substitution matrices, describing the likelihood of an amino acid change, were used to generate molecular features of physicochemical and evolutionary characteristics [64–67].

### Statistical analysis

Statistical analysis was performed on all the molecular features generated to investigate the differences between the ccRCC and non-ccRCC causing mutations. Using the Welch Two Sample t-test, all the values of each feature were analyzed to determine any significant differences in mean between the cancer and non-cancer causing mutations (Supplementary Table 5). The Welch Two Sample t-test test was chosen due to the large imbalance between the number of benign and pathogenic mutations in our dataset, as it does not assume that the groups have equal variances and performs well with unequal sample sizes. This analysis was carried out using the *ggsignif* software package for the R programming language. The cut-off for statistical significance of mean difference between the two populations was set to *P*-value < 0.05.

### Feature selection

As part of the machine learning pipeline, greedy feature selection was used in order to decrease feature redundancy, complexity and risk of overfitting in the final model. The greedy feature selection method consisted of initially evaluating each feature independently in their capability for phenotype prediction, determined by the MCC. The MCC was chosen as a performance metric for model evaluation as it accounts for imbalances between classes in our dataset. MCC scores were generated for 10-fold cross validation, and our non-redundant test set. The best performing feature across both datasets is then selected, and combined with the remaining individually, in an iterative manner. This produces a cumulative list of feature combinations with their corresponding MCC values. This ultimately resulted in a greater performing model consisting of a lower number of features. This method was carried out using the *sci-kit learn* v.1.1.2 Python Package.

### Machine learning

Model generation was performed using sci-kit learn v.1.1.2, based on the training dataset, while a non-redundant test set was used to assess model performance and generalization.

The training dataset consisted of data obtained from the Symphony database (n = 121; ccRCC:67; non-ccRCC:54) and the non-redundant test set was manually curated from the literature (n = 92; ccRCC:75; non-ccRCC: 17). This was performed in order to produce a model directly comparable to the Symphony predictor, especially when subjected to an external blind test. The predictive power of the resulting model was assessed by calculating MCC on the test set. This evaluation metric was used due to an unbalanced test dataset, a result of publication bias in the clinical literature, as the likelihood of the publication of a pathogenic variant is far greater than that of a benign one.

During model development, several machine learning algorithms were assessed in their predictive capabilities, including Gradient Boosting, XGBoost, Random Forest, Extra Trees, Gaussian Process, AdaBoost, K-nearest neighbors, Support Vector Machine, Multi-layer Perceptron, Decision Tree, and Logistic Regression algorithms. The resultant models were assessed using MCC under 10-fold cross validation. In order to increase the clinical applicability of our work, the final model was used to predict the ccRCC-risk of each possible VHL missense through an *in silico* saturation mutagenesis approach.

## Author contributions

A.S. performed all data curation, machine learning and analysis, and wrote the first draft of the manuscript. G.T. and C.S. assisted with data curation and initial analysis. Q.P. assisted with feature engineering. D.E.V.P. provided guidance on data curation. S.P. helped supervise the project. D.B.A. designed and supervised all aspects of the project. All authors reviewed the manuscript.

##  

*Conflict of interest statement:* The authors declare no conflict of interests.

## Funding

This work was supported by an Investigator Grant from the National Health and Medical Research Council (NHMRC) of Australia (GNT1174405 to D.B.A.); Supported in part by the Victorian Government’s Operational Infrastructure Support Program.

## Data availability

All data curated and generated in this study is available in the supplementary tables.

## Supplementary Material

supplementary_figure_1_ddad181

supplementary_tables_marked_edit_ddad181
