# Supplementary figures and images for: Characterizing and predicting ccRCC-causing missense mutations in Von Hippel-Lindau disease
Source: Hum Mol Genet. 2023 Oct 26;33(3):224–32. doi: 10.1093/hmg/ddad181 (PMC10800015; doi:10.1093/hmg/ddad181)

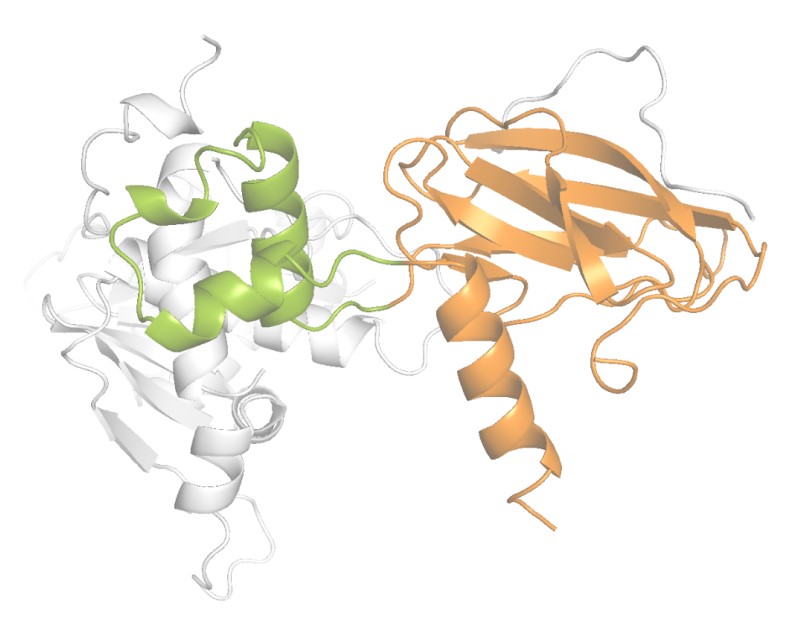

Supplement: supplementary_figure_1_ddad181 [file supplementary_figure_1_ddad181.jpeg]
